# Supplementary figures and images for: Clofoctol inhibits SARS-CoV-2 replication and reduces lung pathology in mice
Source: PLoS Pathog. 2022 May 19;18(5):e1010498. doi: 10.1371/journal.ppat.1010498 (PMC9119441; doi:10.1371/journal.ppat.1010498)

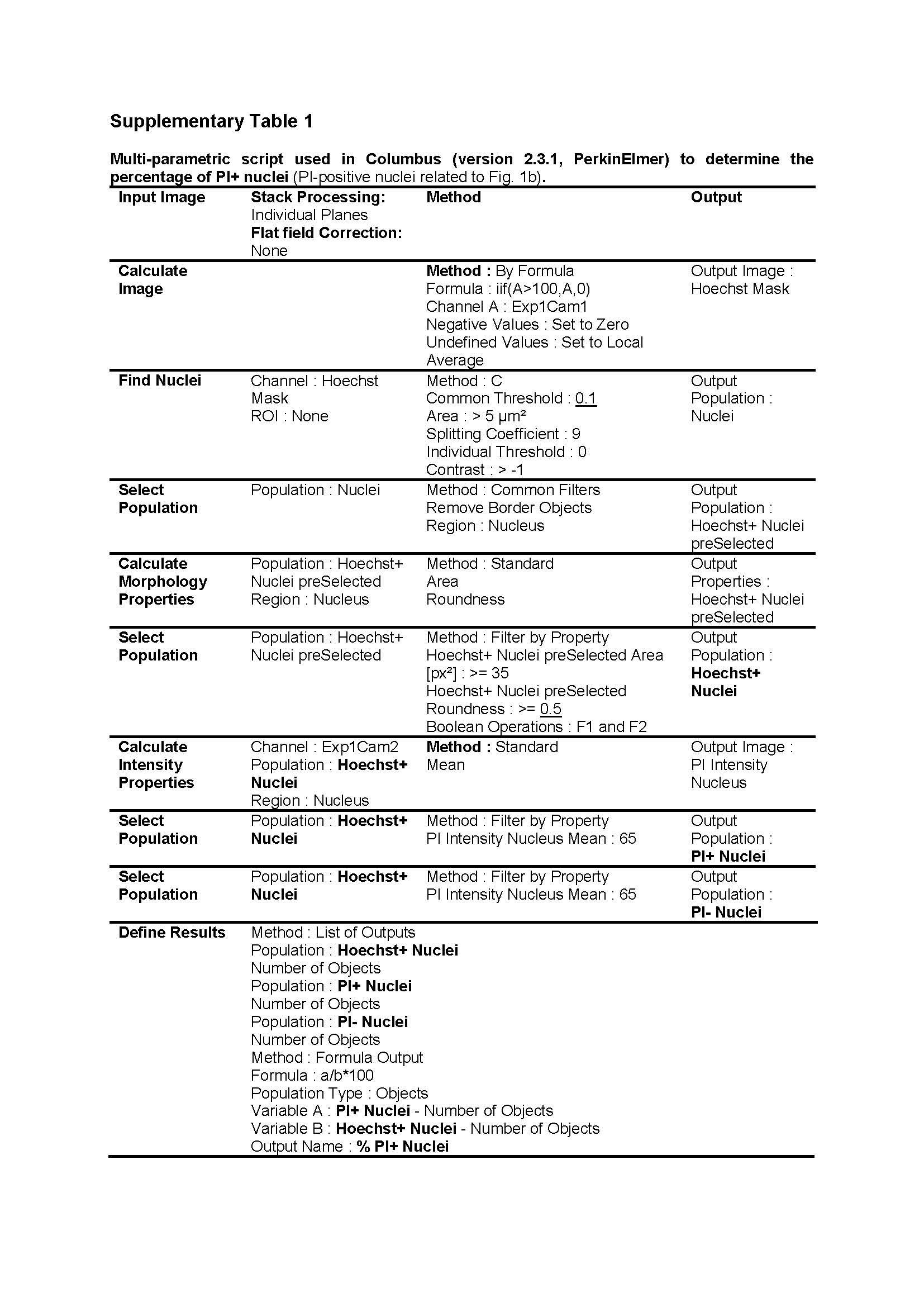

Supplement: S1 Table — (JPG) [file ppat.1010498.s001.jpg]

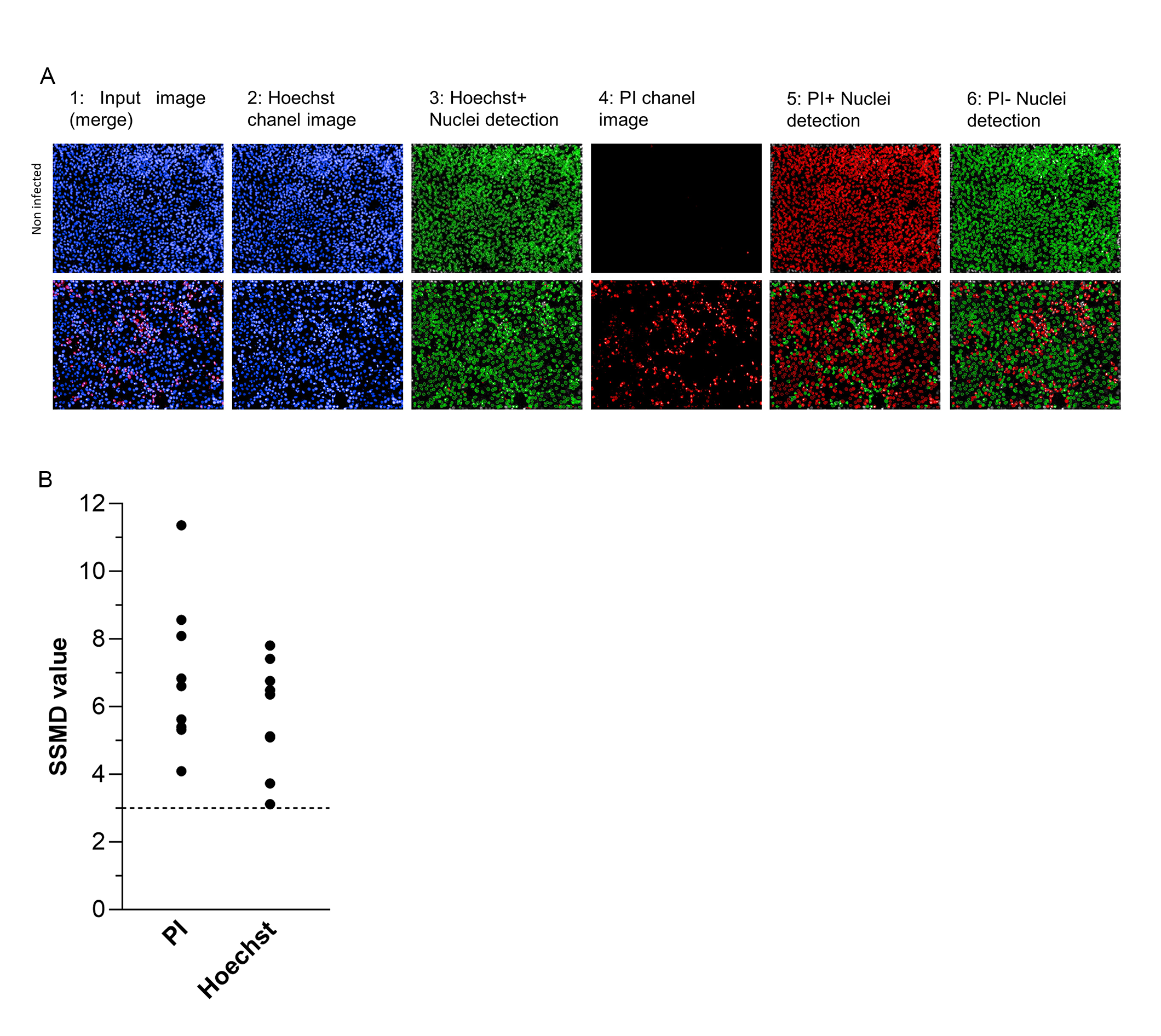

Supplement: S1 Fig — A, Typical images of Vero-81 cells infected with SARS-Cov-2 (top panel: MOI = 0.01) or not (lower panel: Non-infected) acquired on an OPERA QEHS High Content Screening System (PerkinElmer) and corresponding image segmentation. 1: Typical 2-color images (Blue: Hoechst label, Red: PI-label); 2 and 4: 1-color images corresponding respectively to Hoechst and PI channel images; 3: Filled green objects correspond to total segmented nuclei, 5: Filled green objects correspond to PI positive cells or dead segmented cells, 6: Circled green cells correspond to non-infected cells. B, SSMD values for HCS screen of the Apteeus TEELibrary for the identification of anti-SARS-CoV-2 compounds. SSMD values were calculated for all plates by comparing mean and standard deviations on both negative (Mock) and positive (Infected) controls. Dotted-line is indicative of a threshold of 3 allowing for the validation of the plates. SSMDs were calculated for both readouts. (TIF) [file ppat.1010498.s004.tif]

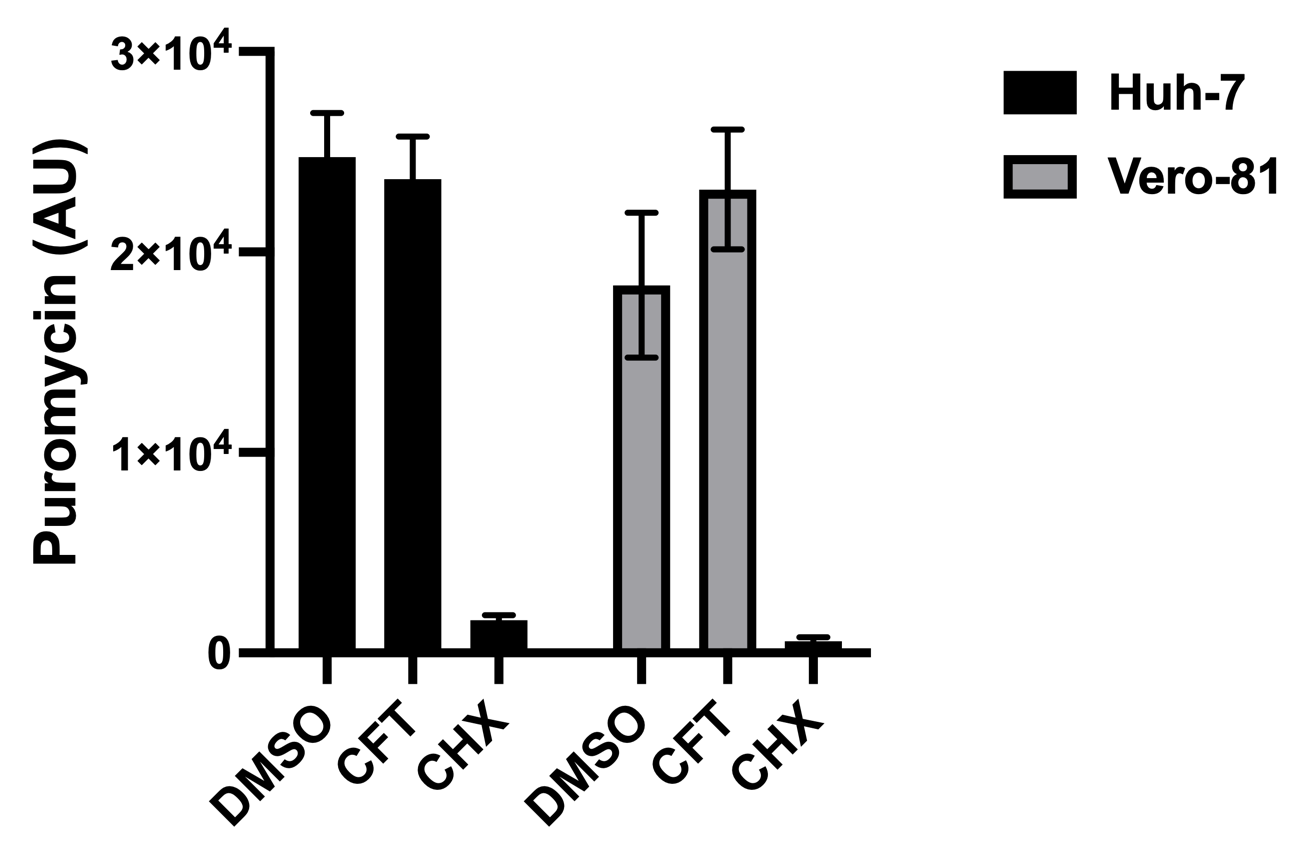

Supplement: S2 Fig — Vero-81 and Huh-7 cells were incubated for 1h with 10 μg/ml puromycin in the presence of 0.05% DMSO, 25 μM clofoctol (CFT) or 100 μM cycloheximide (CHX). Then, the cells were lysed and puromycin-conjugated proteins were quantified by dot-blot with an anti-puromycin antibody. Results show the intensity of the luminescent signal in arbitrary units and represent the average of 4 independent experiments performed in triplicate. Errors bars represent the standard error of the mean (SEM). (TIF) [file ppat.1010498.s005.tif]

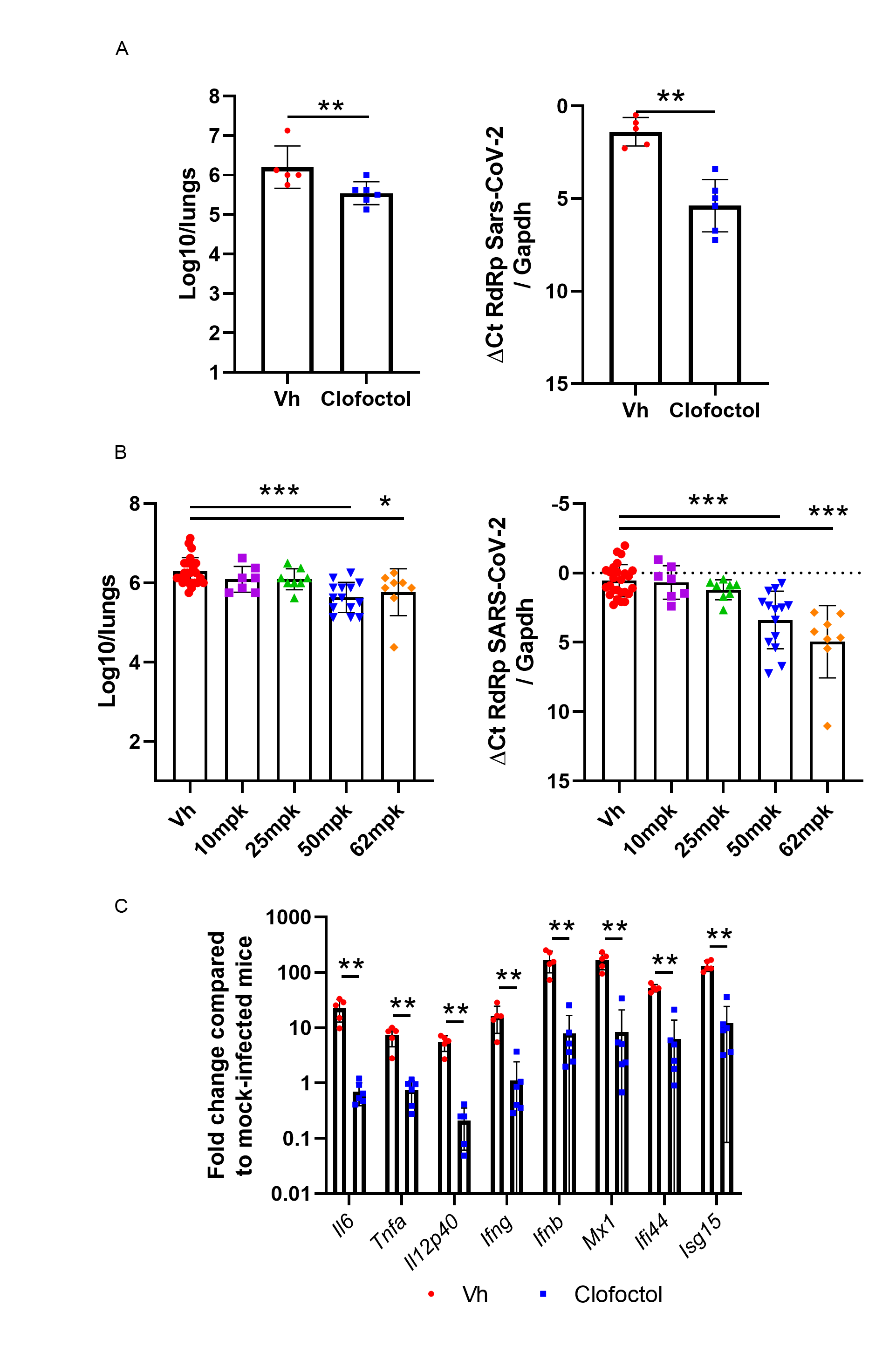

Supplement: S3 Fig — a and b, Male mice were treated (50 mg/kg of clofoctol) and infected as described in Fig 4B. Mice were sacrificed at day 2 post-infection. A, The viral load was determined by titration on Vero-E6 cells (left panel) and by RT-qPCR (right panel). B, The same procedure was applied but with various doses of clofoctol (n = 7–26). C, mRNA copy numbers of genes were quantified by RT-qPCR (50 mg/kg of clofoctol). Data are expressed as fold change over average gene expression in mock-treated (uninfected) animals. Results are expressed as the mean ± SD (n = 5–6). Significant differences were determined using the Mann-Whitney U test (**p < 0.01). (TIF) [file ppat.1010498.s006.tif]

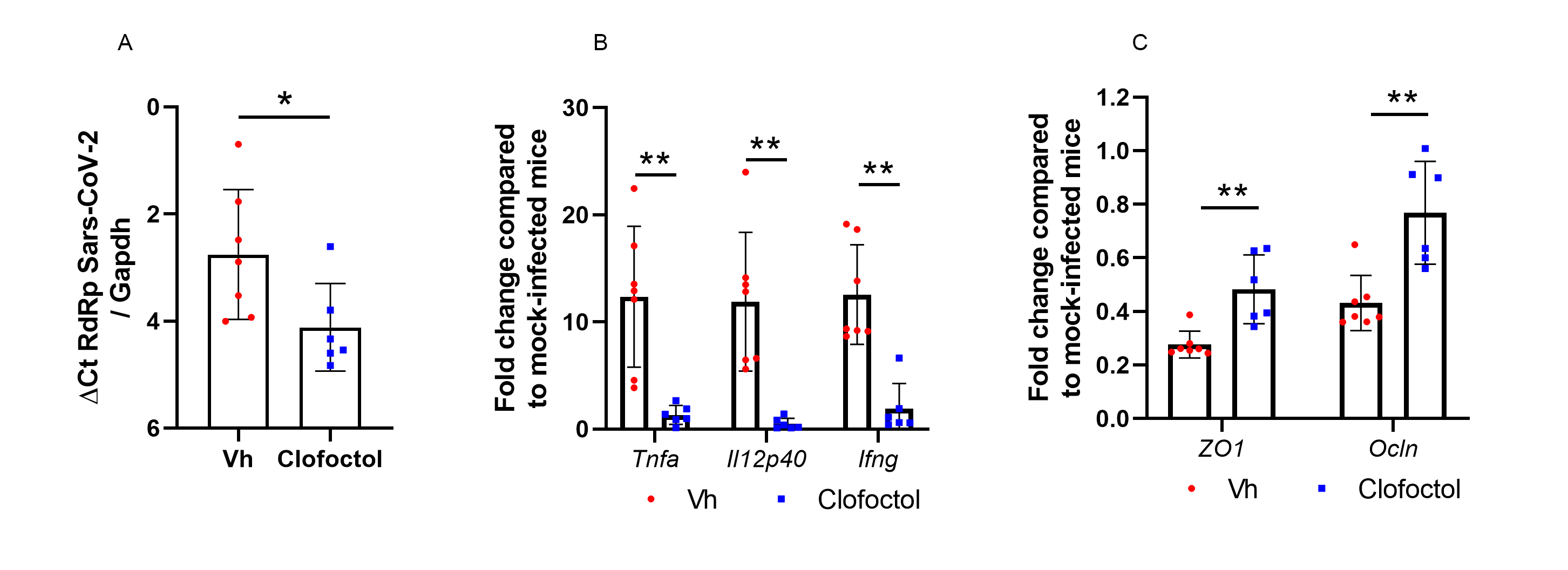

Supplement: S4 Fig — A and B, Female mice were treated and infected as described in Fig 4B. Mice were sacrificed at day 4 post-infection. mRNA copy numbers of genes were quantified by RT-qPCR. Panel B includes inflammatory genes and panel c includes genes involved in barrier function. Data are expressed as fold change over average gene expression in mock-treated (uninfected) animals. Results are expressed as the mean ± SD (n = 6–7). Significant differences were determined using the Mann-Whitney U test (*p < 0.05; **p < 0.01). (TIF) [file ppat.1010498.s007.tif]
