# Supplementary material for: Clofoctol inhibits SARS-CoV-2 replication and reduces lung pathology in mice
Source: PLoS Pathog. 2022 May 19;18(5):e1010498. doi: 10.1371/journal.ppat.1010498 (PMC9119441; doi:10.1371/journal.ppat.1010498)
Supplement: Attachment — Submitted filename: reply-to-reviewers.docx [file ppat.1010498.s008.docx]

**Clofoctol inhibits SARS-CoV-2 replication and reduces lung pathology in mice**

**PPATHOGENS-D-21-02342**

**We would like to thank the editor and the reviewers for their thorough evaluation and fair comments. Our point-by-point responses to the comments received are summarized below, with the reviewer's original question italicized, and our response in bold.**

**Editor**

*Overall, the reviewers were positive in their examination of the manuscript. However, several points need to be addressed and added to. Specifically, reviewers 2 and 3 requested additional in vivo data, including weight loss data from the current experiments. Similarly, the addition of therapeutic treatment or a higher dose of virus would significantly strengthen the manuscript. Based on the relatively low dose, it is unclear how effective clofocotl will be in preventing severe disease following SARS-CoV-2 challenge. Similarly, therapeutic (d0 or d1) treatment would provide insight for utility in treating in humans. More exploration of the specificity to SARS-CoV-2 UTR for targeting is also encouraged. Narrowing to the 5’ or 3’UTR (or both) would strengthen the manuscript and the author should offer discussion on mechanisms of action in the discussion. While one reviewer suggested use of more current variants of interest, this request would be difficult and not necessary for this manuscript. The authors should considering moving in vitro variant data to the main text, however.*

**As suggested by reviewers 2 and 3 additional in vivo data are now provided, including weight loss (see page 7, lines 19-20, page 9, lines 30-36 and page 10, lines 1-5). We have also tested different doses of clofoctol (see Figure S3). Our data show that clofoctol induces weight loss in mice which is attributed to a decrease in gastric emptying. This is a phenomenon specifically observed in mice since it is not observed in human beings, and this limits our capacities to further explore clofoctol in the mouse model. Concerning the SARS-CoV-2 UTR, a paragraph has now been added in the discussion section (page 9, lines 10-18). Finally, an additional variant (delta) has also been tested and the data have been moved to the main text as recommended (See page 6, lines 14-15 and Figure 4).**

**Reviewer #1**

*Effective therapeutics to counter disease caused by SARS-CoV-2 are still lacking despite the ongoing global pandemic. The authors here describe a pharmacological screen of licensed therapeutics for antiviral activity against SARS-CoV-2. From this screen they identified clofoctol, an antibacterial drug, to have high activity against SARS-CoV-2 in vivo and in vivo using a human ACE2-expressing mouse model. The design and robustness of the screening process was of high quality and included numerous biologically relevant controls to identify clofoctol as having antiviral properties regardless of SARS-CoV-2 entry route. In vitro, they demonstrate a reduction of infectious virions produced in the absence of cytotoxicity following simultaneous treatment with clofotol. Its activity again other coronaviruses and variant spike proteins from SARS-CoV-2 indicate its broader applicability as a therapeutic for coronavirus disease. Further, the authors observe a slight drop in viral load and decreased inflammatory gene expression in SARS-CoV-2-infected K18-hACE2 when treated with clofoctol. The manuscript is clearly written, the approach is appropriate, and the findings are relevant.*

**We thank the reviewer for these positive comments**

*The following major critiques and suggestions would significantly improve the manuscript:
1. For figure 3a, when adding drugs at the various steps, it’s not clear why adding Remdsivir before virus infection has no effect. One would imagine, if it is still present at the time of infection and post-entry it would still have its effect. This is seen in figure 3b where Remdesivir is added at 0hr or 1hr. Is this lack of inhibition observed in 3a because the drug is washed off the cells during or just after infection? This is not clear from the legend and should be made explicit.*

**We apologize for the lack of precision in the legend of Fig 3a. The compounds were actually only present during the indicated steps, therefore remdesivir when added during the pre-incubation was washed off and was no longer present during inoculation and the post-infection step. In figure 3b, when the remdesivir was added at 0 and 1h, it was present until the end of the experiment. The legend of the figure is now modified to clarify this point: “Vero-81 cells were infected with SARS-CoV-2 at an MOI of 0.25, clofoctol, remdesivir or CQ were present at a concentration of 15 µM either before infection, during virus entry, post-inoculation or throughout the steps as indicated on the schematic depiction of the experiment. Bars indicate when the drugs were present during the experiment for each condition.” The exact protocol is also detailed in the material and methods (See page 13, lines 26-28).**

*2. It seems odd that addition of clofoctol increases IRF1b Fluc activity but not the SARS-UTR luc activity? Is there a hypothesized mechanism for sequence selective translational inhibition? Later in the discussion, it is mentioned that clofoctol was shown to inhibit protein translation to impair tumor growth. It would be helpful to show that global protein translation is not inhibited in cells treated with this drug, which would otherwise indicate a cytostatic activity of this drug and imply alternative mechanisms of viral inhibition.*

**IRF1b-Fluc activity was increased in Huh-7 cells but we did not observe an increase in Vero-81 cells. Concerning the potential effect of clofoctol on global protein translation, we used a puromycin-conjugation assay adapted from Schmidt et al. (Nature Methods, 2009), which allows to measure the global effect on translation. Cells were incubated in the presence of puromycin with clofoctol or cycloheximide (as a positive control of translation inhibition). We did not detect any difference in protein synthesis as revealed by puromycin-conjugation in clofoctol treated cells as compared to the DMSO control. In contrast we did not detect any puromycin-conjugated protein in cycloheximide treated cells, indicating that clofoctol does not have a global effect on protein synthesis. These data have been added to the manuscript (see page 6, lines 28-32 and Supplementary Figure S2; See also change in the Methods section page 14, lines 32-36 and page 15, lines 1-6).**

*3. Difference in dCt RdRp over Gapdh at day 2 between clofoctol-treated vs vehicle treated mice is not maintained at day 4. This suggests that the treatment may slow viral spread early but does not completely inhibit viral spread. This observation calls into question the in vivo effectiveness of this drug to reduce the burden of infection, although the observed decrease in inflammation may reduce disease. This should be discussed by the authors.*

**We agree with the reviewer on this comment. The effect is reduced at 4 dpi as compared to 2 dpi. The lower effect of clofoctol on day 4 is due to the fact that mice were only treated for 2 days. This is now indicated in the revised version of the manuscript (See page 7, lines 33). This short duration of treatment is due to the fact that we could not treat the mice for longer periods due to the fact that clofoctol induces weight loss which is attributed to a decrease in gastric emptying. However, this does not affect the potential use of this compound in the clinic since this phenomenon is not observed in humans. This differential effect is probably due to the mode of administration which is different in humans (suppository instead of i.p.). This point is mentioned in the discussion section of the revised manuscript (page 9, lines 30-36 and page 10, lines 1-5), in addition to answer to similar concern of reviewer #3.**

**Minor issues**

*The following minor critiques would improve the manuscript:*

*1.For simple comparison of translation inhibition by clofoctol in the two cell lines in Figure 3d, the y-axis range of both graphs should be consistent.*

**The y-axis range is now the same for both graphs.**

*2. Why are only female mice presented in figure 4? If log virus production was similar in male and female, why not pool them? Choice to separate and bury male data in the supplemental is confusing.*

**Data from sexes combined is shown in document 1. For the article, we think it is important to separate the data as experimental conditions are different (50 mpk in male versus 65 mpk in female).**

**Document 1: Antiviral properties of clofoctol in a mouse model of COVID-19**. Female and male K18-hACE2 transgenic C57BL/6J mice were treated i.p. with clofoctol (62.5mg/k for female and 50mg/kg for male) or vehicle 1h and 8h after i.n. inoculation of SARS-CoV-2 (5x10^2^ TCID_50_ per mouse) and treated again twice at day 1 post-infection. Animals were sacrificed at day 2 post-infection. The viral load was determined by titration on Vero-E6 cells. The viral load in female (n=13) and male (n=5-6) mice have been pooled. Results are expressed as the mean ± SD. Significant differences were determined using the Mann-Whitney U test (***p < 0.001).

*3. Unsure what relevance the rectal administration of clofoctol referenced in the discussion. Are the authors suggesting this would be a preferred route of administration for covid-19 patients? Have oral routes of administration been studied?*

**Rectal administration is not the preferred route but the only one that has been developed so far for this drug in humans.**

**Reviewer #2:**

*Belouzard et al. perform a drug screen for in their ability to inhibit SARS-CoV-2 in Vero-81 cells and obtain several hits, including the now defunct chloroquine, a parent compound of hydroxycloroquine. The investigators then focus their study on the bacterial antibiotic clofoctol. While it is unclear if these studies in mice will ultimately translate to humans and the treatments against SARS-CoV-2 remain limited and thus this study is of overall interest. Belouzard and colleagues report potentially interesting findings. However, several major points need to be appropriately addressed before this manuscript can be accepted:*

*1. The investigators examined the antiviral activity of clofoctol in Vero-81 and in Calu-3 cells. The investigators should also examine if clofoctol can inhibit SARS-CoV-2 and specifically a relevant variant such as B.1.617.2 (delta) or B.1.1.529 (omicron) in primary human airway epithelial (HAE) cells. Currently, they investigators have only examined clofoctol against a SARS-CoV-2 isolate from June of 2020 and variants no longer relevant in Vero-81 and Vero-81 TMPRSS2 cells.*

**As suggested by the reviewer, we have also tested clofoctol against the delta variant of SARS-CoV-2 and a similar antiviral activity was observed against this variant (see Fig. 3). We also agree that ALI culture of HAE is a good pre-clinical model for SARS-CoV-2 infection. We actually tested the effect of clofoctol on Mucilair^TM^ but we encountered difficulties with this model. We actually obtained inconsistent results in this model due to the hydrophobicity of this compound which has a tendency to form micellar structures which do not easily reach the basolateral face of the epithelium, when added in the basolateral chamber. Furthermore, administration from the apical pole was also difficult to control. The kinetics of infection in HAE are also very long (72h in our assay), therefore, the cells are probably not correctly exposed to the clofoctol during the whole duration of the experiment. Still, we did confirm the antiviral effect of clofoctol in Calu-3 cells that originates from human lung adenocarcinoma and it was also validated in the K18-hACE2 mouse model.**

*2. The investigators examine the therapeutic efficacy of clofoctol in the K18 mouse model using an early SARS-CoV-2 isolate. Notably, the effect of clofactol on SARS-CoV-2 replication is minimal. The middle panel B in Figure 4 only shows a little over 1 log in viral titer reduction in the lungs of K18 mice. The y axis should be adjusted to not overinterpret the data and should start at Log10 (1) and shown all the way to 7 – axes need to be adjusted. The investigators should also perform a clofoctol therapy mouse study using a relevant variant (either delta or omicron) in addition to the early SARS-CoV-2 isolate.*

**As proposed by the reviewer, we have amended the Figure 4c (See Fig 5c of the revised version) and the Supplementary Figure S4a. We have also provided the data regarding the effects of clofoctol on the delta variant *in vitro* (See Fig 3). Since the antiviral effect of clofoctol is very similar against all the variants tested, we do not think that there is a need to investigate the *in vivo* effect of clofoctol upon SARS-CoV-2 delta variant infection.**

*3. The K18 model is being underused and the investigators should examine if clofactol also can improve mouse survival – the investigators should perform a survival study and take the mice through day 8 or later and infect with a higher dose such as 1X10^4 or 5X10^4. The investigators are notably using a very low dose of 5X10^2 of an early SARS-CoV-2 isolate (Genbank: MW575140) and should evaluate if clofoctol can inhibit replication and mitigate disease against at least one relevant SARS-CoV-2 variant such as delta or omicron.*

**The K18 model is not a perfect model as mice die from encephalitis. This is now mentioned in the discussion section (page 9, lines 30-36 and page 10, lines 1-5). Concerning the dose of inoculation, we would like to mention that it is lethal in mice at 7-8 post-infection. It is indeed mentioned in the manuscript that a lethal dose of virus was used in the in vivo experiments (See page 7, line 16). Therefore, we believe that there is no need to enhance the infectious dose. Furthermore, as mentioned by Reviewer #3 (comment 3), it is clear that the dose used (in our study) is sufficient to induce inflammation and viral replication within the lung.**

**As required, we examined the potential effect of clofoctol on mouse survival. Unfortunately, in our setting, clofoctol did not improve the survival outcome (document 2). This is now mentioned in the discussion section. We have also discussed the potential reasons explaining this effect (page 9, lines 30-36 and page 10, lines 1-5).**

**Document 2: Effect of clofoctol treatment on mouse survival**. Male K18-hACE2 transgenic C57BL/6J mice were treated i.p. with clofoctol (50mg/kg) or vehicle 1h and 8h after i.n. inoculation of SARS-CoV-2 (5x10^2^ TCID_50_ per mouse) and treated again twice at day 1 post-infection (n=8/group).

*4. It is unclear why the authors chose 50mg/kg to treat male mice and 62.5mg/kg in females? Was a PK study performed to inform these doses?*

**As shown in Figure 4A, a PK study was performed on female mice at 62.5 mpk. Regarding the males, we decided to administer the same quantity of clofoctol, regardless the body weight of the mice (20g for females, 25g for males). This is the reason why data shown in Supplementary Fig. S3 correspond to 50 mpk. Please note that we also tested clofoctol at 62.5 mpk in male mice. These data are now shown in Supplementary Fig. S3b.**

*In follow up in vivo experiments, the investigators should determine if there is a dose-dependency in vivo? Can the authors pick one mouse sex and perform a low dose, a middle dose (e.g., 50 or 62.5mg/kg) …..*

**As requested, we have investigated the effect of clofoctol at 10 mpk and 25 mpk. At these doses, and compared to 50 mpk, clofoctol is less efficient in terms of virus load. These data are now shown in new Supplementary Fig. S3b (please also see document 3 for the weight curves).**

*…..and a high dose to examine if there is a dose-dependent viral inhibition by viral titer readout/immune dampening?*

**While it is much better tolerated in humans (suppository), clofoctol induces weight loss in mice, which is attributed to a decrease in gastric emptying. Please note that at 62.5 mpk (female) or at 50 mpk (male), mice lost a significant body weight as early as day 2 post-infection (dpi). This is the reason why (i) no higher doses were tested in the current study and (ii) treatment was stopped at 2 dpi (document 3). The effect of clofoctol on weight loss is now shown in the new Fig. 5b (*right* panel). This is important to mention and we thank the reviewer for this comment.**

**Document 3: Effect of varying doses of clofoctol on body weight loss**. Male K18-hACE2 transgenic C57BL/6J mice were treated i.p. with various doses of clofoctol or vehicle 1h and 8h after i.n. inoculation of SARS-CoV-2 (5x10^2^ TCID_50_ per mouse) and treated again twice at day 1 post-infection. Weight loss was measured until day 2 post-infection (n=8/group).

*5. The investigators examine the viral step at which clofoctol can inhibit SARS-CoV-2 and determine a post entry step and specifically translation, by the inhibition or a reporter controlled by 5’ and 3’ UTR of SARS-CoV-2. Did the authors also sequence virus at day 4 post infection in the K18 mice?*

*Are there, for reasons that may be unexpected/incompletely understood, selection of SARS-CoV-2 mutants arising in K18 mice treated with clofoctol compared to infected mice which are untreated with clofoctol?*

**This is an interesting question. However, in our experimental setting, the animals were treated with clofoctol only for 2 days. So, the lower effect of clofoctol at day 4 is likely due to the absence of treatment after day 2. We are aware that it would be interesting to determine the appearance of resistant mutants which would further help to better understand the mechanism of action of this molecule. However, this is beyond the scope of this paper.**

**Minor issue**

*Figure 3D needs an accurate label – it should read “SARS-CoV-2-UTR” or “SARS2-UTR”. Currently, the way the label is written refers to SARS-CoV, the virus that caused the 2003 outbreak.*

**The Figure has been modified as suggested.**

**Reviewer #3**

*Overall, this is a very nice study by Belouzard et al. with promising results for the use of clofoctol in the treatment of SARS-CoV-2. The authors identified clofoctol in a screen of 1,942 drug candidates for inhibition of CPE in Vero81 cells induced by SARS-CoV-2 infection. The IC50 of clofoctol was determined for several different cell types, and the authors show that clofoctol inhibits SARS-CoV-2 through inhibition of viral translation through a series of in vitro experiments. Importantly, the authors demonstrate antiviral efficacy of clofoctol in vivo in a K18-hACE2 mouse model of infection, showing a significant reduction in viral burden in the lungs as well as reduced induction of pro-inflammatory cytokines and inflammation in clofoctol-treated animals. The major strengths of the study are 1) the comprehensive validation approach of the drug candidates, which include analysis of both TMPRSS2-dependent and independent entry in multiple different cell lines; 2) the identification of clofoctol as a potential therapeutic, which has not been studied to date; 3) the finding that clofoctol inhibits SARS-CoV-2 viral translation; and 4) in vivo demonstration of antiviral and anti-inflammatory impact in the K18-hACE2 mouse model. I particularly liked that the authors used live virus to perform their screen and used multiple measures of viral replication (CPE-based microscopy, RT-PCR of SARS-CoV-2 RdRp, western blot of N-levels, luciferase constructs, and TCID50), which contributes to the robustness of their findings.*

**We would like to thank this reviewer for these positive comments.**

*Can the authors include any weight loss data that was collected for the animal studies for both the infection K18-hACE2 experiments and the pharmacokinetics? This data would be helpful to further assess and support the impact of clofoctol on SARS-CoV-2 infection as well as safety and tolerability of the drug.*

**We fully agree with this comment. Data are now shown in Fig. 5b (*right* panel). Also, as required by reviewer #2, a dose-dependence response is provided. See also answer to comment 3 of reviewer #1.**

*2. The in vivo data presented in Figure 4 demonstrates both an impact on viral replication and inflammation. While it can be difficult to unlink the impact on viral replication with induction of inflammation, the authors raise the possibility in the discussion of an anti-inflammatory role of clofoctol that may be independent from the impact on viral replication, which could be doubly useful in the treatment of severe disease when viral replication and spread may be in decline. Have the authors pushed administration of clofoctol until after peak viral load in the lungs (2-4 dpi) to determine if clofoctol may also work to dampen inflammation at these later time points?*

**This is a very interesting suggestion. However, as mentioned in the answers to questions of the two other reviewers, we had to stop the treatment after two days because clofoctol induces weight loss which is attributed to a decrease in gastric emptying, a phenomenon that is not observed in humans. Currently, our objective is to find another formulation and/or route of administration to treat mice without inducing weight loss. As suggested, this might improve efficacy as indicated in the revised version of the discussion (page 9, lines 30-36 and page 10, lines 1-5).**

*3. The dose for the infection appears to be on the lower end of the range of infection doses given to K18-hACE2 mice in studies published by others (5 x 102 TCID50 compared to 2.5 x 103 PFU; 2.5 x 104 PFU; 103-104 TCID50). That being said, it is clear that the dose used is sufficient to induce inflammation and viral replication within the lung. Have the authors tested the efficacy of clofoctol following higher infection doses?*

**Virus titers in lungs of infected animals do not always correlate with the dose of the virus inoculum. In our experimental conditions, the virus load at 2 dpi is identical whatever the dose inoculated (5 x 10^2^ TCID50 and 5 x 10^3^ TCID50) (document 4). As previously discussed, the inoculum used in our experiments (5 x 10^2^ TCID50) is sufficient to kill mice and this explains why the potential efficacy of clofoctol was not tested at higher inoculating viral doses.**

**Document 4: Dose-dependent inoculating viral dose on viral load in lungs.** Female K18-hACE2 transgenic C57BL/6J mice were i.n. inoculated with two different doses of SARS-CoV-2 (5x10^2^ TCID_50_ and 5x10^3^ TCID_50_ per mouse. Animals were sacrificed at day 2 post-infection. The viral load was determined by titration on Vero-E6 cells. Results are expressed as the mean ± SD (n=5-6).

*4. The inclusion of chloroquine and remdesivir as comparative controls that inhibit different steps in viral replication makes it very tempting to compare the efficacy of clofoctol against these two drugs. How do the IC50 of chloroquine and remdesivir in these assays compare with clofoctol?*

**We did not aim at comparing the efficacy of the different compounds that were used as controls in Figure 3. The IC50 of chloroquine and remdesivir in Vero-81 cells are approximately 2 µM and 4.5 µM, respectively. Remdesivir is a prodrug and the efficacy to produce its active metabolite is cell type-dependent. Therefore, the relative potency of the nucleoside prodrug is also cell-type dependent. Chloroquine only inhibits endosomal entry of coronaviruses and our assay was performed in the absence of any TMPRSS2 expression. We used these drugs as their mechanism of action are well-known to discriminate the different steps of viral cycle in cells and we used a concentration higher than the antiviral IC50 to reach pharmacological saturation.**

*5. The authors show that clofoctol also inhibits HCoV-229E and seems to specifically target SARS-CoV-2 transcripts but does not inhibit translation of genes following HCV IRES. Can the authors comment on if there is something specific about coronavirus replication that may confer this specificity?*

**Translation of viral RNA requires interaction of viral RNA with the host cell translational machinery. Several studies have uncovered RNA binding proteins (RBPs) important for infection. It is likely that clofoctol may act on one of these RBPs to inhibit viral translation. Comparison of translational factors recruited by SARS-CoV-2 or flavirirus (ZIKV, DENV) showed different preferences for elongation initiation factors. Indeed SARS-CoV-2 prefers EIF3B, 4H, 4B, 3F, and A3 whereas flaviviruses prefer EIF3A, 4G1, 3C, and 3D (Flynn et al. Cell 2021). More specifically, CSDE1 has been identified as a proviral factor that binds viral RNAs (Lee et al., Mol Cell, 2021).** **This is particularly interesting as clofoctol has been shown to bind CSDE1 (Hu et al. JCI, 2019). However, it is not known if CSDE1 binds the UTR region or another region of the viral RNA. This discussion has now been added to the manuscript (See page 9, lines 10-18).**

**In addition, the translation efficiency of viral RNAs compared to cellular RNA differs between viruses. Therefore, is it likely that interactions between the viral RNA and the host machinery are specific for a virus family. In addition, it has been shown that the coronavirus nsp1 inhibits the host cell protein translation and viral infection may have virus-specific consequences for the host cell translation machinery.**

**Minor issue**

*1.The authors perform a nice set of experiments to define where in the viral replication cycle that clofoctol is inhibiting SARS-CoV-2. The details regarding the first set of experiments described (Figure 3a) are missing, and specifically the time points that correspond to each step that the authors are aiming to capture.*

**For clarity, we changed the legend of the Figure to indicate that bars in the schematic depiction of the experiment indicate when the compounds are present for each condition. The time points that correspond to each step can be found in more details in the material and methods section (1h for the pre-incubation; entry is composed of two steps of one hour: one for the inoculation and one for completing endocytosis and fusion; for the post-entry, the compounds are added after the entry step until the fixation of the cells)(See page 13, lines 26-28).**

*2. How expensive is a current regimen of Octofene/GramPlus? Is it a feasible clinical option for treatment of infection in resource poor settings? I think that a brief discussion of these factors would help the reader understand the feasibility of this potential therapeutic.*

**The current price of a 5-day treatment at the adult posology of 0.75g bid, rectally is less than 15€. The current market size of Gramplus is small, but a significant reduction in the cost of treatment is expected from a significant increase in the scale of production. In addition of being a drug of real potential in resource poor settings, its economical affordability and very good tolerance profile in humans could allow prescription to a large population of patients (at risk or not), very early in the course of the disease, to reduce viral load in the respiratory tract and, expectedly reduce human to human disease transmission. A sentence has been added in the introduction (See page 3, lines 9-11) and at the end of the discussion (See page 10, lines 19-21).**
